# RETRACTION: Green synthesised zinc oxide nanostructures through *Periploca aphylla* extract shows tremendous antibacterial potential against multidrug resistant pathogens

**DOI:** 10.1049/nbt2/9813605

**Published:** 2026-09-08

**Authors:** IET Nanobiotechnology

RETRACTION: F. Abbas, Q. Maqbool, M. Nazar, et al., “Green synthesised zinc oxide nanostructures through *Periploca aphylla* extract shows tremendous antibacterial potential against multidrug resistant pathogens.” *IET Nanobiotechnology*, 11 (2017): 935–941, https://doi.org/10.1049/iet-nbt.2016.0238.

The above article, published online on 10^th^ August 2017 in Wiley Online Library (wileyonlinelibrary.com), has been retracted by agreement between the Journal Editor‐in‐Chief; The Institution of Engineering and Technology; and John Wiley & Sons Ltd.

The retraction has been agreed due to concerns raised by a third party. An investigation revealed that images in Figure 3 were previously published elsewhere in a different scientific context by a partially overlapping author group. When the authors were contacted for an explanation, they stated that they would need additional time for gathering all the necessary data. They were given a timeline to provide the same but have not responded. Accordingly, we cannot vouch for the integrity or reliability of the content and have taken the decision to retract the article. The authors have been informed about the decision to retract.

